# Dasatinib Attenuates Pressure Overload Induced Cardiac Fibrosis in a Murine Transverse Aortic Constriction Model

**DOI:** 10.1371/journal.pone.0140273

**Published:** 2015-10-12

**Authors:** Sundaravadivel Balasubramanian, Dorea L. Pleasant, Harinath Kasiganesan, Lakeya Quinones, Yuhua Zhang, Kamala P. Sundararaj, Sandra Roche, Robert O’Connor, Amy D. Bradshaw, Dhandapani Kuppuswamy

**Affiliations:** 1 Cardiology Division of the Department of Medicine, Gazes Cardiac Research Institute, 114 Doughty Street, Charleston, South Carolina, United States of America; 2 Dublin City University, Dublin 9, Ireland; Texas A& M University Health Science Center, UNITED STATES

## Abstract

Reactive cardiac fibrosis resulting from chronic pressure overload (PO) compromises ventricular function and contributes to congestive heart failure. We explored whether nonreceptor tyrosine kinases (NTKs) play a key role in fibrosis by activating cardiac fibroblasts (CFb), and could potentially serve as a target to reduce PO-induced cardiac fibrosis. Our studies were carried out in PO mouse myocardium induced by transverse aortic constriction (TAC). Administration of a tyrosine kinase inhibitor, dasatinib, via an intraperitoneally implanted mini-osmotic pump at 0.44 mg/kg/day reduced PO-induced accumulation of extracellular matrix (ECM) proteins and improved left ventricular geometry and function. Furthermore, dasatinib treatment inhibited NTK activation (primarily Pyk2 and Fak) and reduced the level of FSP1 positive cells in the PO myocardium. *In vitro* studies using cultured mouse CFb showed that dasatinib treatment at 50 nM reduced: (i) extracellular accumulation of both collagen and fibronectin, (ii) both basal and PDGF-stimulated activation of Pyk2, (iii) nuclear accumulation of Ki67, SKP2 and histone-H2B and (iv) PDGF-stimulated CFb proliferation and migration. However, dasatinib did not affect cardiomyocyte morphologies in either the ventricular tissue after *in vivo* administration or in isolated cells after *in vitro* treatment. Mass spectrometric quantification of dasatinib in cultured cells indicated that the uptake of dasatinib by CFb was greater that that taken up by cardiomyocytes. Dasatinib treatment primarily suppressed PDGF but not insulin-stimulated signaling (Erk versus Akt activation) in both CFb and cardiomyocytes. These data indicate that dasatinib treatment at lower doses than that used in chemotherapy has the capacity to reduce hypertrophy-associated fibrosis and improve ventricular function.

## Introduction

Cardiac fibrosis is one of the detrimental factors that contributes to heart failure during increased cardiac workload under conditions such as hypertension or aortic stenosis. Increased accumulation of fibrotic proteins within the myocardium, especially in the interstitium and in perivascular areas has been implicated in the progression of heart failure [1–5]. In the injured myocardium, collagen deposition in response to myocyte loss is a reparative process; however the loss of homeostatic balance of ECM remodeling and extracellular accumulation of ECM proteins leads to an increased accumulation of collagen. The resultant reactive fibrosis contributes to increased stiffness, electrical impedance and diastolic dysfunction in the heart. In addition, paracrine factors that are secreted by pro-fibrotic fibroblasts are often detrimental to the function of cardiomyocytes [6, 7]. Thus, in a tissue environment undergoing adaptive remodeling in response to an increased myocardial workload (hypertrophy in this case), attenuation of the mitogenic/profibrotic and inflammatory signaling processes, specifically in the fibroblast population might improve heart function. Currently there are limited options to treat cardiac fibrosis. Therefore, newer approaches at the molecular level are needed to address this problem. In this context, identification of specific pathways that promote the mitogenic, secretory and proliferative potential of cardiac fibroblasts (CFb) may serve as a unique target for treating cardiac fibrosis. An attractive approach is to utilize specific anticancer drugs that block cancer cell proliferation, invasion and tissue fibrosis. However, it has been shown that several anticancer drugs exhibit cardiotoxic effects in a subset of patient populations [8], although the underlying mechanisms remain largely unknown. We have recently reported that CFb from β3-/- integrin mice induced with hypertrophic stimulation exhibited a low fibrotic status (as seen by reduced collagen and fibronectin accumulation in the ECM) in the myocardium [9]. This indicates that β3 integrin might mediate mitogenic and proliferative signaling in CFb of PO myocardium. Because integrins have no intrinsic enzymatic activity, these receptors primarily recruit specific nonreceptor tyrosine kinases (NTKs) to mediate downstream signaling. In line with this idea, previous studies have shown that Src family NTKs might be potential targets for antifibrotic therapy [10, 11]. Therefore, to explore whether blocking NTK activation in pressure overloaded (PO) myocardium suppresses cardiac fibrosis, we used dasatinib, a clinically administered FDA approved anticancer drug. Similar to imatinib, dasatinib is a newly developed tyrosine kinase inhibitor that targets c-abl and c-kit; however, dasatinib also inhibits PDGFR and Src family tyrosine kinases. Dasatinib treatment in skin fibroblasts obtained from systemic sclerosis patients responded positively with reduced ECM synthesis and extracellular deposition [12]. A clinical trial (NCT00764309) to study the safety of dasatinib is ongoing in subjects with scleroderma pulmonary fibrosis. These earlier and ongoing studies on dasatinib prompted us to explore whether this drug treatment could alleviate cardiac fibrosis in PO myocardium. In the present study, we used dasatinib at a low concentration and showed that dasatinib treatment during both PO *in vivo* and in cultured CFb *in vitro*, substantially reduced ECM deposition. Our studies also show that dasatinib treatment blocked NTKs such as Pyk2 and Fak and was found to reduce both proliferation and migration of CFb. Finally, dasatinib treatment not only reduced ECM deposition but also improved ventricular function and geometry during PO, indicating that this drug used at appropriate lower doses may offer therapeutic benefits to patients with chronic PO.

## Materials and Methods

### Reagents

Dasatinib was obtained from LC Laboratories. Antibodies used were from the indicated vendors: Pyk2 (BD Biosciences), phospho 402 Pyk2, phospho Src 416, phospho Fak 925, phospho histone H2B, phospho Erk, phospho Akt 473 (Cell Signaling), Ki 67 (Abcam), GAPDH (Fitzgerald), collagen, fibronectin, vinculin (Santa Cruz), alpha-actinin (Sigma), FSP1 (Millipore) and SKP2 (Abcam).

### Adenoviruses

Adenoviral construct for the expression of dominant negative c-Src (double mutant, K295R and Y527F) was generated using He's pAdEasy-1 system [13] as described previously [14, 15]. Fak-CD (c-terminal domain) adenoviral construct was generously provided by Dr. William Cance’s laboratory. Adenoviral constructs for the expression of kinase inactive mutants of Pyk2 (Y402F and Y457F) were generously provided by Dr. Roland Baron, Harvard University.

### Mice

C57BL/6 wild type (WT) mice were obtained from Jackson Laboratories and the colony was maintained at the Medical University of South Carolina (MUSC) animal care facility. All animal studies were conducted in accordance with the Guide for the Care and Use of Laboratory Animals (National Research Council, National Academy Press, Washington, DC, 1996) and were approved by the Institutional Animal Care and Use Committee at MUSC (Approval ID: ACORP443).

### Transverse aortic constriction (TAC)

Pressure overload was induced in adult mice (three months old male mice, weighing approximately 30 g) as reported previously [16] by tying a suture around the transverse aorta over a 27-gauge blunted-needle causing occlusion of the aorta. The needle was withdrawn, resulting in a stenotic aortic lumen. Four weeks after TAC surgery, animals were euthanized by removal of the heart in deep anesthesia. Sham-operated mice without TAC served as controls.

### Echocardiography

For mouse echocardiography, the Vevo2100 imaging system (VisualSonics, Toronto, Canada) with 22–55 MHz linear transducer probe (MS550D) was used for two-dimensional B-mode and M-mode analyses. Heart rate was maintained at 400–500 bpm via isoflurane anesthesia. The mitral valve leaflet was visualized and its function was assessed at long axis B-mode view by placing the transducer on the left lateral chest wall. End-systolic and end-diastolic LV dimensions and wall thicknesses were measured according to the American Society of Echocardiography guidelines as applied to mice [17]. LV wall thickness was measured at the level of intraventricular septum and the posterior wall. LV volume was calculated using Simoson’s method of disks and ejection fraction determined using the formula: LV end-diastolic volume (EDV) − end-systolic volume (ESV) /LV end-diastolic volume (EDV). Offline image analyses were performed using dedicated VisualSonics Vevo2100 1.2.0 software.

### Dasatinib delivery

Mice were anesthetized under surgical isoflurane anesthesia (5% induction; 2–3% maintenance) and placed on a warming blanket. The thoracic region was cleaned with isoprpopanol and a small incision was made ventrally near the intraperitoneal cavity (i.p). The Alzet pump (Alzet model #1004, Durect Corp, Cupertino, CA; 100 μl capacity) was inserted into the i.p. cavity as we performed previously [18], and the incision was repaired. The pump was implanted either two days prior or two weeks after the TAC and sham surgery which allowed us to study the effect of dasatinib prior to the initiation of pressure overload or after establishing cardiac hypertrophy. We used i.p. instead of subcutaneous route for the drug delivery, since these mice were subsequently used for TAC or Sham surgery. The pump (100 μl volume) contained 0.5 mg dasatinib dissolved in 50% DMSO in saline and released the solution at a rate of 0.11 μl/hour to deliver dasatinib at 0.44 mg/kg/day. For control mice, a mini pump containing only the vehicle (50% DMSO) was used. Mice did not show any internal injuries in the i.p. area during the slow release of either drug or the vehicle. Upon termination of the experiments, the mice were anesthetized with 5% isoflurane balanced with O_2_, and the heart was removed for histochemical and biochemical studies. In addition, the i.p. cavity was monitored for signs of injury.

### Primary cell isolation and culture

Primary CFb were isolated from 3-month old mouse hearts (male or female) as reported previously [19]. Briefly, hearts were removed, rinsed in PBS, minced, and subjected to collagenase digestion in 1:10 diluted Blendzyme-3 (Roche, Indianapolis, IN) in DMEM (Invitrogen) at 37°C for 1–3 h. Tissue was triturated, and the resulting cell suspensions were rinsed three times in growth media (DMEM containing 10% fetal bovine serum (FBS) and antibiotic-antimycotic solution) before final plating. All experiments with CFb were performed between passages 2 and 3 in serum free or 10% FBS containing media. For cardiomyocytes, adult feline hanging heart preparation using enzymatic digestion was adopted and cardiomyocytes were cultured in serum free media as per the protocols described previously [20].

### Immunohistochemistry

At the end of the PO duration, left ventricular tissue was removed and freshly frozen in OCT compound or fixed in 4% paraformaldehyde at room temperature followed by washing in PBS for subsequent paraffin embedding. For collagen volume fraction (CVF), hearts were fixed in formalin and stained with picrosirius red as described. CVF was determined in five fields from three separate animals from each group as described previously [9, 21]. For immunostaining, fresh frozen sections were permeabilized with 0.1% Triton X-100 and then blocked with 10% normal donkey serum for 1 h at room temperature. The sections were then incubated with primary antibodies (1:500 dilutions) for overnight at 4°C. Following three washes in PBS, secondary antibodies conjugated to Alexa Fluor dyes (1:1000 dilutions) and DAPI (1:1000 dilution for nuclear staining) were incubated for 2 h at room temperature. The slides were then washed three times in PBS, mounted with coverslips using Mowiol and subjected to laser scanning confocal microscopy (Olympus IX81).

### Immunocytochemistry

CFb or cardiomyocytes grown or plated on coverslips were fixed with 2% paraformaldehyde, permeabilized with 0.1% Triton X-100 and then blocked with 10% normal donkey serum for 1 h at room temperature. The primary antibodies (1:500 dilutions) were then incubated with the coverslips for overnight at 4°C. Following three washes in PBS, secondary antibodies conjugated to Alexa Fluor dyes (Life Technologies; 1:1000 dilutions) and DAPI (1:1000 dilution) were incubated for 2 h at room temperature. The coverslips were then washed three times in PBS, mounted onto glass slides using Mowiol and subjected to laser scanning confocal microscopy (Olympus IX81).

### Western blotting

Cells or ventricular tissue samples were extracted using Triton X-100 containing buffer to obtain soluble proteins and processed with SDS sample buffer as described previously [22]. Proteins in SDS sample buffer were resolved by SDS-PAGE and transferred to PVDF membranes. The membranes were blocked for 1 h using 1% BSA and 5% milk in TBST (10 mM Tris, 0.1 M NaCl, 0.1% Tween-20, pH 7.4). Blots were incubated with primary antibodies in TBST overnight at 4°C, washed five times, each for five minutes with TBST, and then incubated with horseradish peroxidase conjugated secondary antibody in TBST for 1 h at room temperature. After five washes, each for 5 minutes with TBST, the proteins were detected by enhanced chemiluminescence (PerkinElmer, Wellesley, MA).

### Migration assay

CFb migration was measured by using Oris Cell Migration Assay kit as per the manufacturer’s instructions (Platypus Technology) and as we described previously [9]. Cell migration was induced with PDGF-BB (PDGF, 10 ng/ml). After 28 h, the cells were fixed with 4% paraformaldehyde and stained with phalloidin-Alexa Fluor 568 and DAPI and analyzed by fluorescence microscopy at 10X (Olympus IX71).

### Proliferation assay

^3^H-thymidine incorporation assays were performed as reported early [9, 23]. CFb were plated at equal densities in 24-well plates (6 wells per condition) and allowed to adhere overnight. The cells were stimulated by the addition of PDGF (10 ng/ml) for 18 h and then incubated with 2 μCi/ml ^3^H-thymidine (6.7 Ci/mmol; Amersham, Arlington Heights, IL) for 4 h. The following protocol was used to measure ^3^H-thymidine incorporation: cells were 1) rinsed two times in cold PBS, 2) 10% trichloroacetic acid added for 30 min at 4°C, 3) washed in cold 100% ethanol, 4) solubilized in 0.1 N NaOH for 30 min at 65°C, and 5) radioactivity measured in a scintillation counter.

### Cellular Uptake of Dasatinib

Uptake of dasatinib by cardiomyocytes and CFb was determined as described earlier [24]. Briefly, cells were treated with 100 nM of dasatinib for 60 min. Cells were washed thoroughly in PBS and collected by scraping. To the cell pellet, 150 μl of ammonium formate (1 M, pH 3.5) was added, reconstituted and transferred to a 10 ml extraction tube. Fifty microliters of ammonium formate was added to the original eppendorf tube and spun briefly in order to transfer the remaining traces of cell material to the extraction tube.

One hundred microliters of internal standard (lapatinib) and 1.6 ml of extraction solvent (*t*BME/ACN, 3/1 v/v) were added to the cell pellet. The samples were mixed and centrifuged and 1.1 ml of the supernatant was removed and allowed to evaporate. After reconstituting in 40 μl of acetonitrile, samples were analyzed by LC-MS. Analysis was performed in MRM mode with the following transitions: m/z 581 → m/z 365 for lapatinib, and m/z 488 → m/z (231 and 401) for dasatinib where 401 m/z was the quantifier ion. Peaks were quantified using Agilent Masshunter Software. Quantification was based on the peak area of dasatinib quantifier ion (488→401 m/z) and the peak area of lapatinib (ISTD) 581→365 m/z [24].

A standard curve was prepared based on the LOG10 of the spiked drug versus the LOG10 of the peak area ratio (dasatinib/lapatinib). The LOG10 of the PAR of the sample was substituted into the equation and the mass was back calculated. The mass was normalized to the cell number and the protein mass and the triplicates were averaged. Finally, the quantity of dasatinib was expressed as ng/pl based on cell volumes for cardiomyocytes and CFb.

### Statistics

Statistical comparisons among groups were performed using one-way ANOVA followed by a Tukey post hoc test for echocardiography measurements. Student's unpaired *t*-test was performed for fluorescence image quantification. Statistical significance was defined as p<0.05.

## Results

### In vivo administration of dasatinib ameliorates ECM deposition and improves left ventricular function in TAC mice

**Fig 1:**
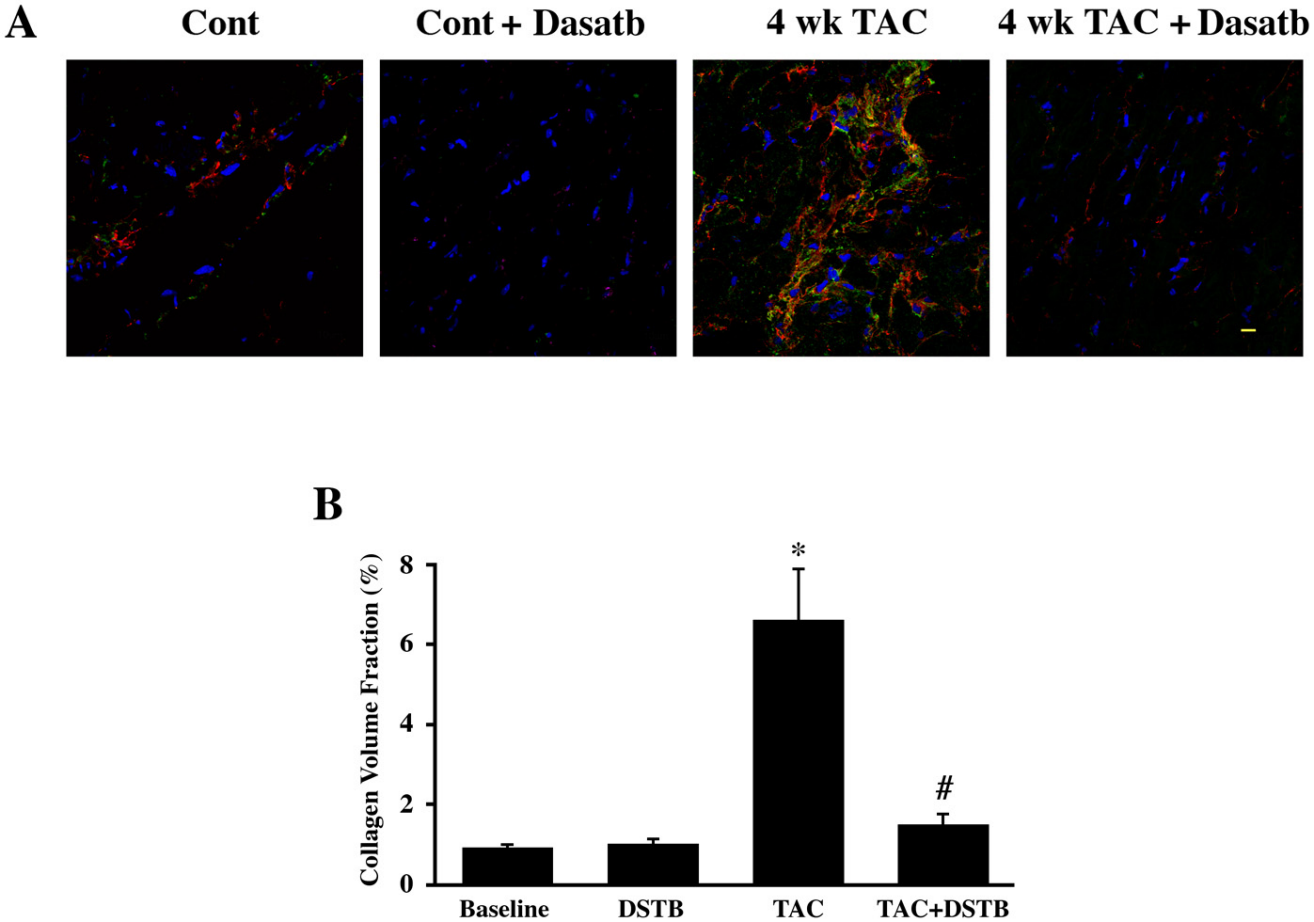
Reduction of pressure overload induced fibronectin and collagen accumulation and maladaptive ventricular changes in dasatinib treated mice. Mice were surgically implanted with osmotic mini-pumps to deliver either vehicle or dasatinib (0.44 mg/kg/day). Two days after mini-pump implantation, mice were used either for TAC or sham (Control) surgery. After 4 wk, echocardiography was performed and end points taken. (**A**) LV sections were stained for fibronectin (red) and collagen-1 (green) using specific antibodies and nuclei using DAPI (blue). Results were confirmed in two additional mice samples (n = 3) for each group. *Scale bar*, *10 μm*. (**B**) LV sections for each mice group (n = 6) were stained for collagen using picrosirius red and the collagen volume fraction was calculated. *p< 0.05 vs. Control; #p< 0.05 vs. TAC. (**C**) LV/bodyweight (mg/g) ratio was calculated for all mice after sacrifice (n = 6 for each group) and graphed. *p< 0.05 vs. Control; #p< 0.05 vs. TAC.

Elevated activities of receptor and nonreceptor tyrosine kinases have been linked to cancer and organ fibrosis and anti-cancer drugs have been shown to have the ability to reduce organ fibrosis [11, 25, 26]. In the present work, we explored whether administration of dasatinib, which is used to treat patients with chronic myeloid leukemia (CML), can suppress PO-induced cardiac fibrosis. In CML patients, dasatinib is given at a dose of 100–140 mg/day and is reported to exert a low level of cardiotoxicity [27, 28]. The average body weight of these patients is considered to be 70 kg, hence the above dose corresponds to 1.4–2 mg/kg/day. In the present study, we administered dasatinib at a dose of 0.44 mg/kg/day for the drug treated mice group while the control group received vehicle only. If bodyweight alone is taken into consideration, this dose for the mouse is about four fold lower than the human dose. However, converting the dose for mice requires the inclusion of the body surface area in addition to body weight. Based on the guidelines described earlier [29], the dose used in humans is multiplied by a factor of 12.3 to obtain the corresponding dose for mice, which is 17.5–24.6 mg/kg/day. Comparatively, our studies used a ~50-fold lower dose (0.44 mg/kg/day) than that used for patients. To study the effect of dasatinib during the entire TAC period, the drug was administered via implantation of a mini osmotic pump in the mice two days prior to TAC or sham surgery and the drug delivery was maintained during the 4 wk TAC period. LV tissue sections from the above groups of mice were analyzed for immunohistochemical analysis (Fig 1A). PO by TAC was found to substantially increase the accumulation of fibronectin and collagen, which was markedly reduced in dasatinib treated mice. Measurement of collagen volume fraction using picrosirius red staining was performed for ECM quantification (Fig 1B). A significant increase in collagen volume fraction was observed in 4 wk TAC mice when compared to controls. This increase was not noticeable in the dasatinib treated PO mice indicating that the hypertrophy-induced accumulation of fibrillar collagen in the interstitium and in the perivasculature was largely reduced by dasatinib treatment.

To explore whether the reduction of PO-induced ECM deposition was accompanied by improved ventricular function, gravimetric and echocardiographic measurements were performed (Table 1). Gravimetric analyses revealed a significant increase in LV/BW ratio in the vehicle treated TAC group when compared to sham control group. However, this increase was significantly reduced in dasatinib treated mice. In 4 wk TAC mice, M-mode echocardiography showed a significant increase in LV internal diameter at end systole and diastole, LV wall thickness, end-diastolic volume (EDV) and end-systolic volume (ESV) when compared to the baseline levels. These changes were significantly reduced in the dasatinib treated mouse group. Furthermore, the 4 wk PO caused a significant reduction in the ejection fraction (EF) and fractional shortening (FS) of the vehicle only group. Only the loss of EF was significantly recovered in the dasatinib treated group. Importantly, although most of the changes in PO myocardium were significantly reversed by dasatinib, they were not completely reversed to the baseline levels and remained significantly altered even after dasatinib treatment. Finally, dasatinib treatment alone, in the absence of TAC did not exhibit any significant alterations in the parameters studied, implying that dasatinib treatment does not result in any noticeable toxicity at the doses studied. Furthermore, our TAC surgical protocol generally results in ~30% initial mortality due to surgical trauma. In mice with dasatinib administration, neither the initial nor the overall mortality rate during the 4 wk TAC period was altered (data not shown).

**Table 1:** Echocardiographic measurement of LV geometry and function at 4 wk TAC with or without dasatinib (DSTB) treatment.

	BW	LVW	LVW/BW	WTh	LVIDd	LVIDs	EDV	ESV	EF	FS
	(g)	(mg)	(ratio)	(mm)	(mm)	(mm)	(μl)	(μl)	(%)	(%)
**Control**	**28.92**	**101.5**	**3.52**	**0.82**	**3.53**	**2.33**	**55.18**	**18.03**	**67.57**	**34.07**
(n = 6)	± 1.11	2.64	± 0.09	± 0.01	± 0.02	± 0.1	± 1.14	± 2.09	± 3.35	± 2.4
**DSTB**	**29**	**102.8**	**3.56**	**0.85**	**3.495**	**2.09**	**56.52**	**13.28**	**73.86**	**38.91**
(n = 4)	± 1.44	± 2.02	± 0.12	± 0.01	± 0.024	± 0.05	± 2.11	± 0.51	± 0.94	± 1.41
**TAC**	25.21 *	190.3 *	7.57 *	1.12 *	3.98 *	3.41 *	78.89 *	52.76 *	33 *	14.24 *
(n = 4)	± 0.98	± 3.99	± 0.19	± 0.01	± 0.03	± 0.06	± 1.54	± 2.86	± 4.04	± 2
**TAC+DSTB**	**28.95 ^**	152.3 *^	5.31 *^	1.03 *^	3.8 *^	3.11 *^	68.67 *^	37.98 *^	45 *^	18.28 *
(n = 6)	± 1.07	± 9.43	± 0.39	± 0.03	± 0.06	± 0.1	± 3.38	± 3.35	± 3.48	± 1.88

Age matched C57 mice were subjected to Sham or 4 wk TAC surgery. Echocardiography was performed at 4 wk time point of TAC. DSTB treatment was initiated prior to surgery as detailed in the Methods section.

BW, body weight; LVW, Left ventricular weight; WTh, LV wall thickness; LVIDd, LV internal diameter at end diastole; LVIDs, LV internal diameter at end systole; EDV, end diastolic volume; ESV, end systolic volume; EF, ejection fraction; FS, fractional shortening.

Data are reported as mean ± SEM. * = p<0.05 vs. baseline; ^ = p<0.05 vs. TAC.

### Dasatinib blocks the activation of Pyk2 and Fak and reduces cell proliferation during in vivo pressure overload

**Fig 2:**
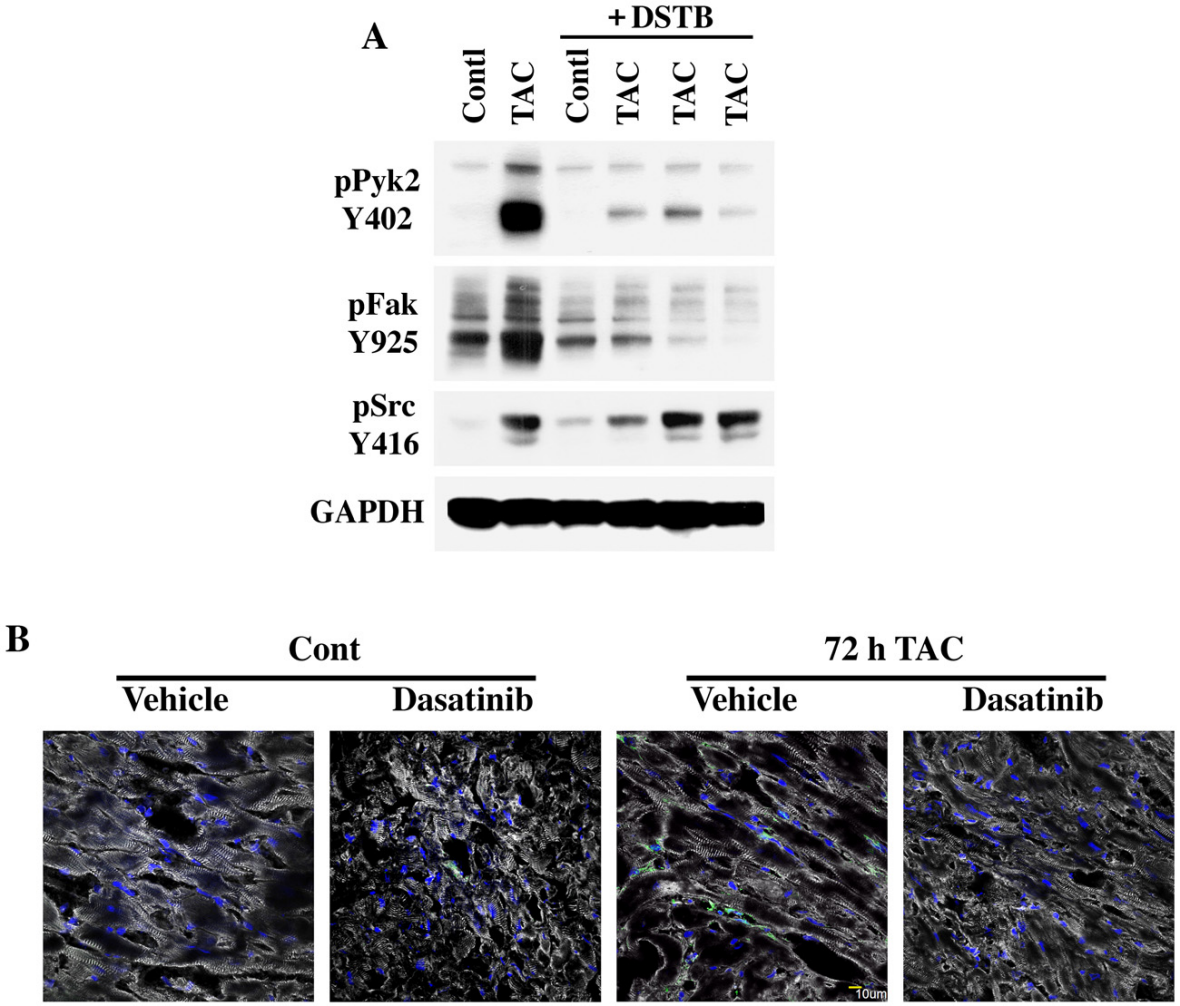
Loss of pressure overload induced nonreceptor tyrosine kinase activation and cell proliferation in LV samples of dasatinib treated mice. Mice were implanted with mini-pumps to deliver either vehicle or dasatinib at 0.44 mg/kg/day for 5 days. Two days after the implantation, they were used either for TAC or sham (control) surgery. After 3 days, hearts were collected and LV tissue samples were processed for Western blot or immunostaining. (**A**) Protein extracts were used for Western blotting with specific antibodies to detect tyrosine-402 Pyk2, tyrosine-925 Fak and tyrosine-416 c-Src. Western blot with GAPDH antibody was used to monitor protein loading. Results were confirmed in two additional experiments (n = 3). (**B**) LV tissue sections were used for immunostaining with anti-α-actinin (Grey) and FSP1 (Green) antibodies and nuclear staining with DAPI (blue). *Scale bar*, *10 μm*.

We next analyzed whether the loss of ECM accumulation in PO myocardium was accompanied by the loss of PO-induced NTK activation. For this, we used a 72 h time point, since our earlier work indicated that the activation of integrin-mediated signaling was primarily observed during 24–72 h of PO [16, 30]. Activation of c-Src, Fak and Pyk2 was detected following a 72 h TAC period (Fig 2A) as evidenced by their phosphorylation state. Dasatinib treatment in the sham control animal did not appreciably change the basal levels of phosphorylation. However, in the dasatinib-treated TAC mouse group, phosphorylation of Pyk2 and Fak was abrogated. However, PO-induced activation of c-Src was not sensitive to dasatinib inhibition. Finally, increased ECM deposition has been observed to associate with increased expression of fibroblast-specific protein-1 (FSP1) [31] and, in our earlier studies, we observed increased levels of FSP1 in PO myocardium [9]. Our present studies show that the level of FSP1 was increased following 72 h TAC and this increase was blocked in dasatinib treated mice (Fig 2B).

### Dasatinib treatment in CFb suppresses ECM deposition and blocks Pyk2 activation

**Fig 3:**
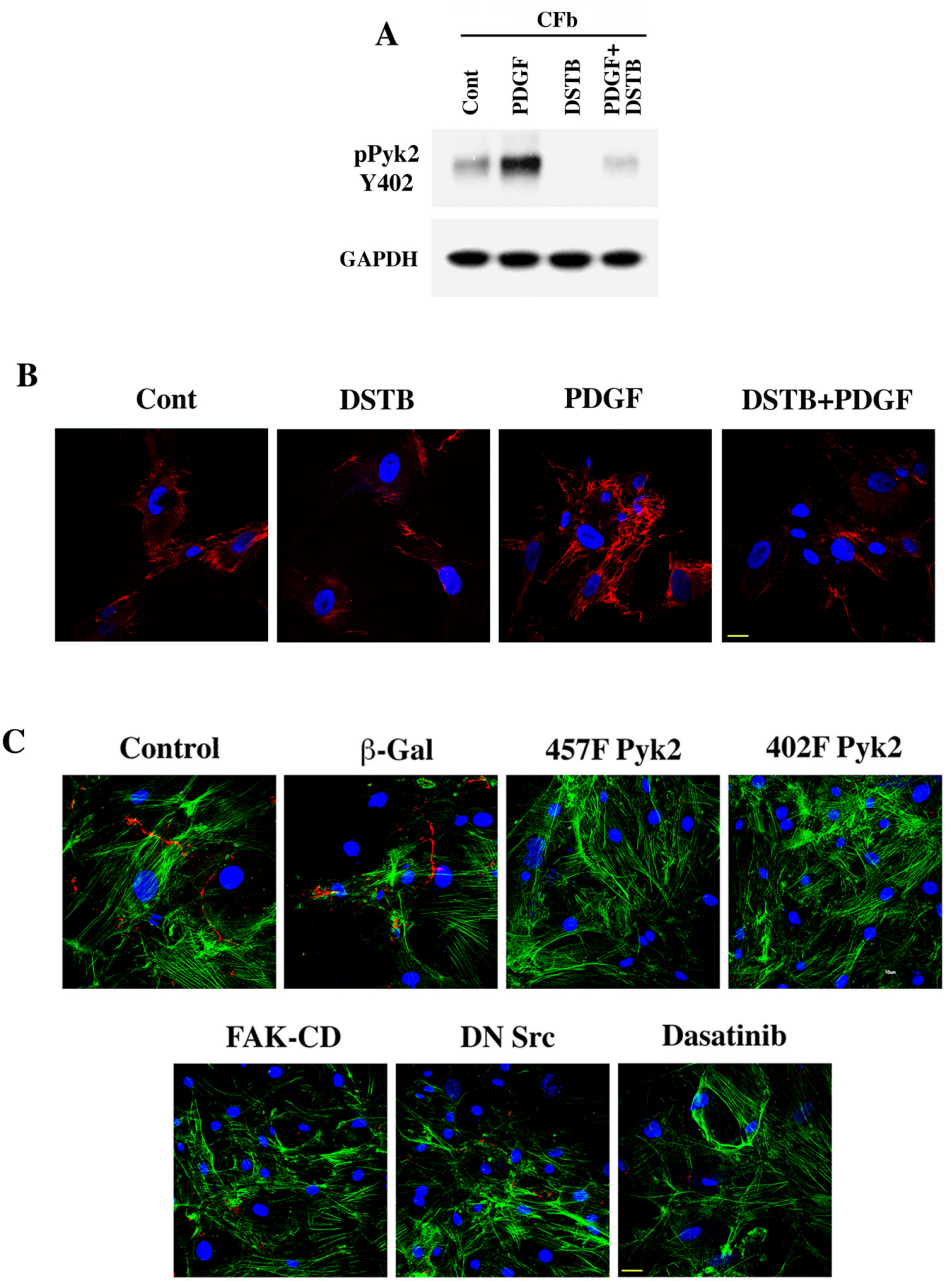
Reduction of PDGF-stimulated fibronectin accumulation and Pyk2 activation in dasatinib treated CFb. **(A)** CFb grown on 35 mm culture plates were pretreated with ± 50 nM dasatinib for 30 min and then stimulated with 10 ng/ml PDGF for 30 min. Triton X-100 soluble proteins were used for Western blot analysis using phospho-tyrosine Pyk2 402 antibody. Western blotting with GAPDH antibody was used to monitor protein loading. (**B**) CFb grown on coverslips were pretreated with ± 50 nM dasatinib for 30 min and then stimulated with 10 ng/ml PDGF for 24 h. After 24 h treatment, cells were fixed and immunostained with anti-fibronectin antibody (red) and nuclear stained with DAPI (blue). Results were confirmed in two additional experiments (n = 3). *Scale bar*, *10 μm*. (**C**) CFb grown on coverslips for 12 h in 10% FBS containing media were infected with adenoviruses for the expression of β-gal (control), Y457F mutant Pyk2, Y402F Pyk2, Fak c-terminal domain, and dominant negative c-Src for 36 h or treated with dasatinib (50 nM) for 24 h. Cells were then used for immunostaining for fibronectin (red) and actin (green) with specific antibodies and for the nucleus (blue) with DAPI. Results were confirmed in one additional experiment. *Scale bar*, *10 μm*.

Next, we explored whether the effects of dasatinib observed *in vivo* on Pyk2 and other NTKs in PO myocardium can be mimicked in isolated CFb *in vitro*. In our recent studies [9], we showed that Pyk2, when compared to Fak and c-Src, was the primary kinase activated during PDGF stimulation and that suppression of Pyk2 activity via adenoviral expression of a mutant form Pyk2 (Y402F) reduced PDGF-stimulated fibronectin assembly. Therefore, to explore whether dasatinib treatment blocks Pyk2 activity and reduces ECM deposition *in vitro*, we cultured CFb overnight in serum free media, pretreated with ± 50 nM dasatinib and then stimulated with ± PDGF. These studies showed that the basal and PDGF-stimulated phosphorylation of Pyk2 at tyrosine 402 (Y402) was substantially reduced (Fig 3A). Furthermore, fibronectin assembly, which was substantially increased in serum-starved cells when stimulated with PDGF, was decreased with dasatinib treatment (Fig 3B). These data indicate that dasatinib may affect ECM deposition by primarily blocking Pyk2 activation. Finally, to demonstrate that Pyk2 is the primary kinase that contributes to ECM deposition, we used several adenoviral constructs to express inactive forms of major NTKs, including Pyk2, Fak and c-Src. For this, we cultured cells in 10% FBS media during the adenovirus infection and analyzed the extracellular accumulation of fibronectin. Compared to β-galactosidase (β-gal) expressing CFb, cells expressing both types of mutant Pyk2 that are known to lack kinase activity showed a strong reduction in fibronectin assembly (Fig 3C). On the other hand, expression of kinase inactive mutants of Fak or c-Src showed a moderate reduction in fibronectin assembly. Importantly, compared to untreated control cells, dasatinib treatment reduced fibronectin assembly, similar to Pyk2 mutants. These studies suggest that Pyk2 functions as a primary NTK responsible for agonist induced ECM deposition and serves as a potential target for dasatinib treatment.

### Dasatinib treatment inhibits nuclear localization of proliferation markers

**Fig 4:**
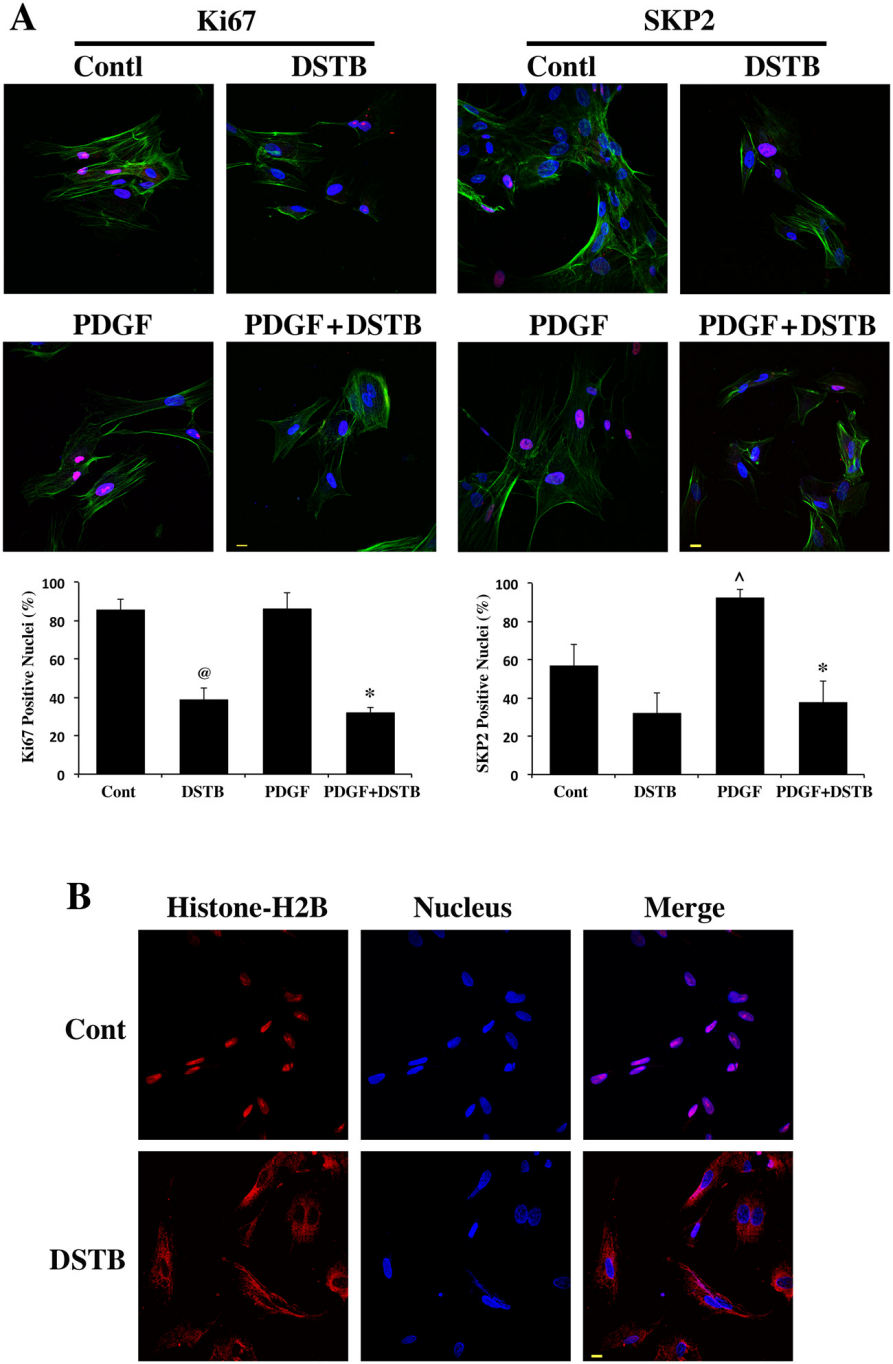
Dasatinib treatment suppresses nuclear localization of proliferation markers. (**A**) CFb were cultured on coverslips for 2 h. For dasatinib treatment, the drug (50 nM) was added to the cells 30 min prior to stimulation with ± PDGF (10 ng/ml). After 24 h treatment, cells were used for immunostaining. Left panels show immunostaining for Ki67 (red) and α-actinin (green) with specific antibodies and nuclear staining with DAPI (blue). *Scale bar*, *10 μm*. *Bottom graph*: quantification of Ki67 positive nuclei was performed by counting over 50 nuclei (DAPI) in multiple sections from two independent experiments. The percentage of Ki67/DAPI is depicted in the graph. @p<0.05 vs. untreated control; *p<0.05 vs. PDGF. Right panels show immunostaining for SKP2 (red) and α-actinin (green) with specific antibodies and nuclear staining with DAPI (blue). *Scale bar*, *10 μm*. *Bottom graph*: quantification of SKP2 positive nuclei was performed by counting over 50 nuclei (DAPI) in multiple sections from two independent experiments. The percentage of SKP2/DAPI is depicted in the graph. ^p<0.05 vs. untreated control; *p<0.05 vs. PDGF. (**B**) CFb were cultured on coverslips for 2 h in 10% FBS containg media and dasatinib (50 nM) or vehicle was then added. After 24 h dasatinib treatment, cells were immunostained for histone-H2B (red) with a specific antibody and nuclear stained with DAPI (blue). *Scale bar*, *10 μm*.

To explore whether the loss of ECM accumulation in dasatinib treated cells was partly due to reduced proliferation of CFb, we analyzed nuclear-bound proliferative markers, Ki67 and SKP2. CFb cultured in serum free media were pretreated with dasatinib for 30 min and then stimulated with ± PDGF. Cells were used for immunostaining. Fig 4A shows the levels of nuclear associated Ki67 and SKP2. As shown in the summary data, compared to the basal (control) levels, PDGF stimulation caused a significant increase in nuclear-bound SKP2 but not in the level of nuclear-bound Ki67. However, dasatinib pretreatment substantially blocked the localization of both Ki67 and SKP2 to the nucleus. We also analyzed the effect of dasatinib on the nuclear localization histone H2B, a critical factor in the nucleus required for cell growth and transformation [32]. For this, studies were done directly in cells cultured with 10% FBS without subjecting them to serum starvation or PDGF stimulation. In the presence of serum, histone-H2B was mostly present in the nucleus (Fig 4B). Interestingly, dasatinib treatment retained histone-H2B exclusively in the cytoplasm (Fig 4B). Together these data indicate that dasatinib affects the spatial distribution of several key components critical for cell proliferation.

### Dasatinib treatment inhibits CFb proliferation and migration

**Fig 5:**
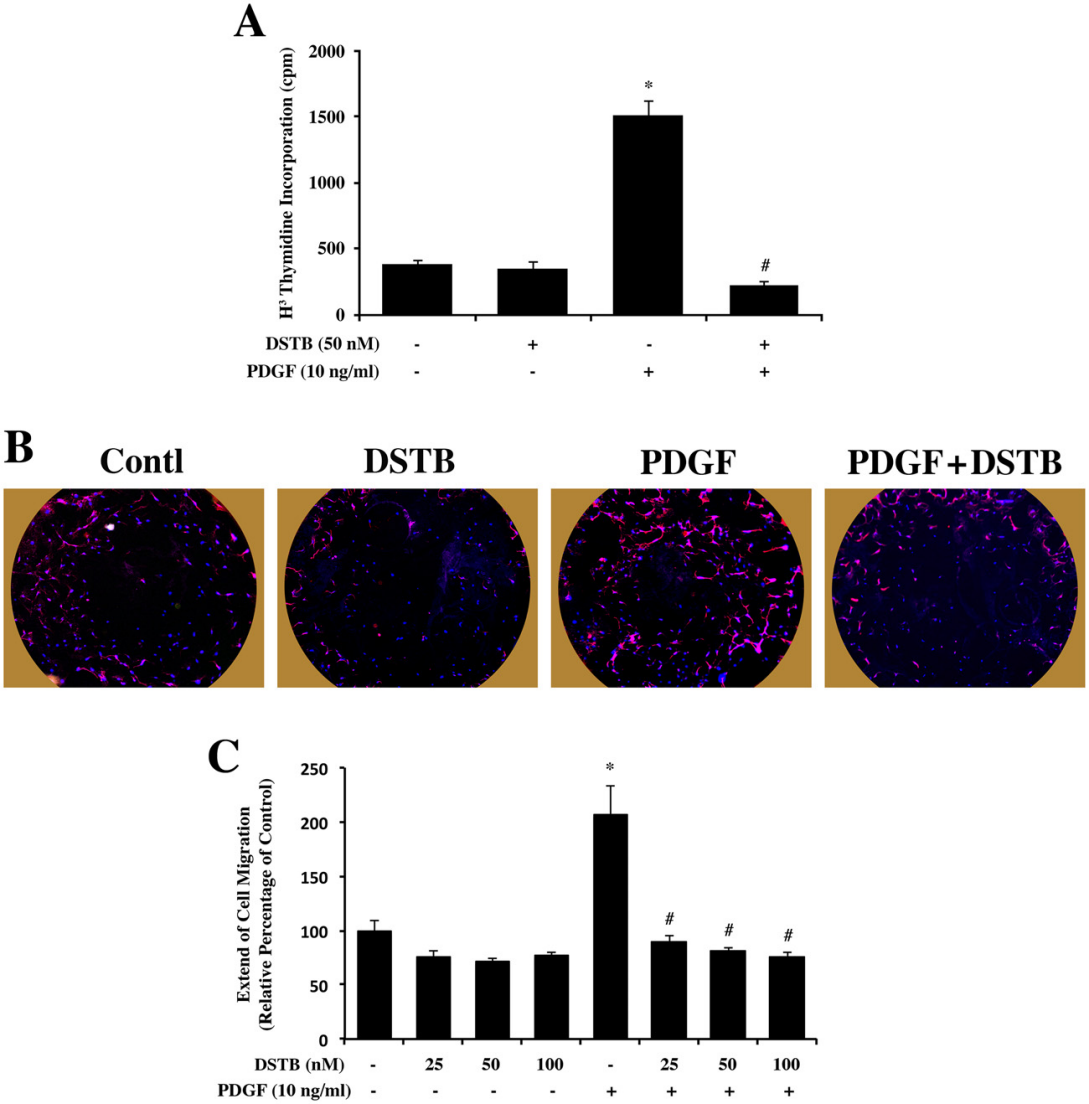
Dasatinib treatment suppresses CFb proliferation and migration. (**A**) CFb cultured on 24 well plates were serum starved for 12 h and then ± 50 nM dasatinib was added 15 min prior to the stimulation with ± PDGF (10 ng/ml). After 18 h treatment, cells were incubated with 2 μCi/ml ^3^H-thymidine for 4 h and processed to measure radioactivity as detailed in the Material and Methods. ^3^H-thymidine data obtained from four independent experiments is shown as mean ± SEM. (**B**) For the cell migration assay, CFb were plated in 96 well format Oris TM plates (Platypus Technology) and allowed to grow overnight. 50 nM dasatinib or vehicle was added to cells and then stoppers were removed. Cells were stimulated to migrate by adding ± PDGF (10 ng/mL). After 24 h, cells were fixed with 4% paraformaldehyde and stained for actin (phalloidin-Alexa Fluor 568; red) and nuclei with DAPI (blue). The entire well was imaged by fluorescent microscopy at 10X. (**C**) Quantification from the migration assay is depicted in the graph where dasatinib treatment was performed at various doses. *p<0.05 vs. untreated control; #p<0.05 vs. PDGF.

To directly study dasatinib effect on CFb proliferation and migration, we performed the thymidine incorporation experiment (Fig 5A). While serum starved cells show low levels of tritiated thymidine incorporation, PDGF treatment resulted in a more than 3-fold stimulation of thymidine incorporation. Importantly, dasatinib treatment blunted the incorporation indicating the PDGF-induced proliferation of CFb was reduced by dasatinib.

Finally, our earlier work showed that the loss of β3 integrin signaling, where NTKs play a downstream role, resulted in the suppression of both CFb proliferation and migration [9]. Therefore, we explored whether dasatinib treatment affected CFb migration in addition to proliferation. For this, we used the Oris Cell Migration Assay as shown in Fig 5B. PDGF-induced CFb migration was significantly reduced when the cells were treated with dasatinib. A dose-dependent decrease in the migration of these cells in response to dasatinib was observed with > 80% reduction at a concentration of 50 nM (Fig 5C).

### Dasatinib induced no major changes in cardiomyocyte gross morphology and/or signaling

**Fig 6:**
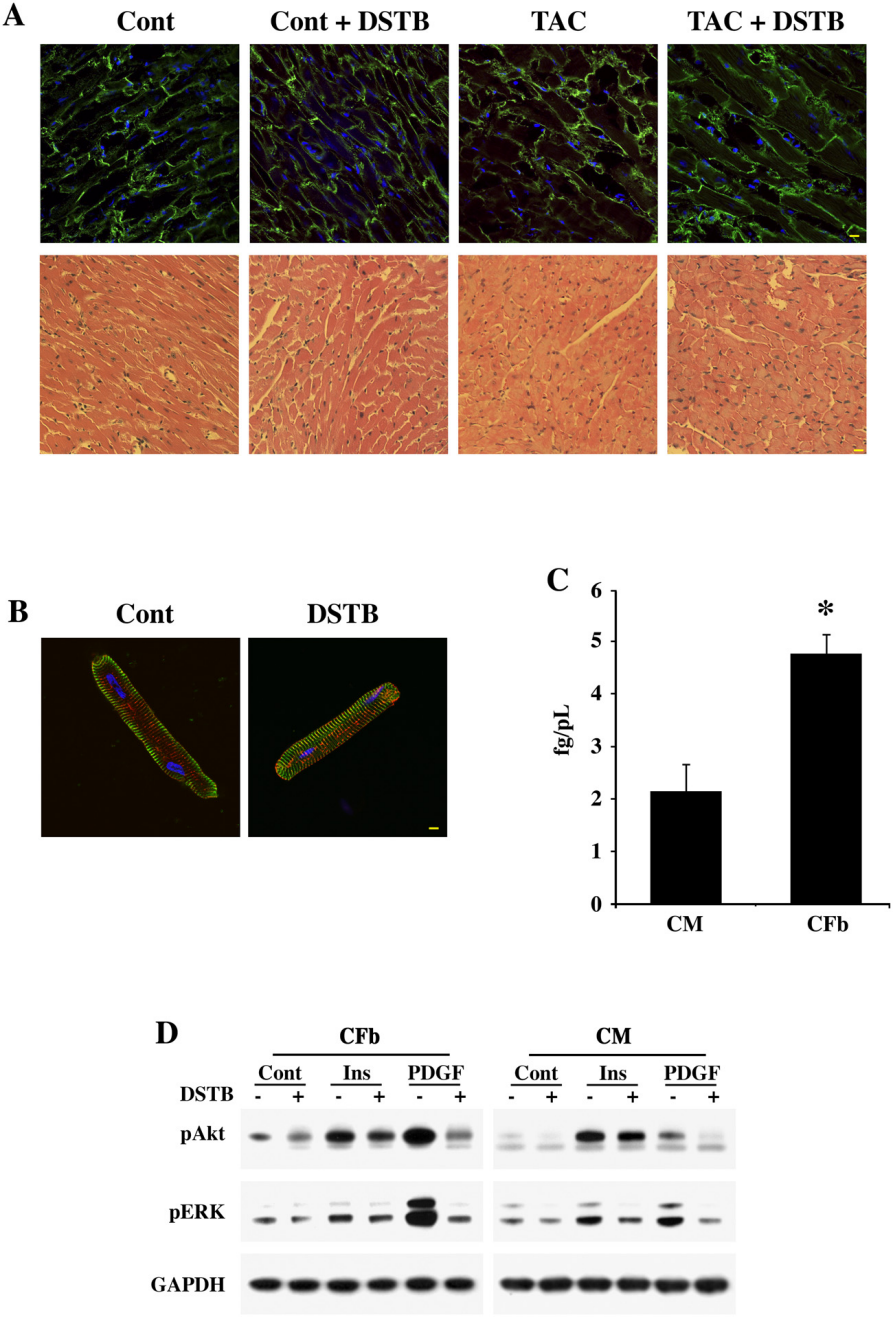
Dasatinib effect on cell morphology and signaling. (**A**) Mice were implanted with mini-pumps to deliver either vehicle or dasatinib at 0.44 mg/kg/day for 30 days. Two days after the implantation, they were used either for TAC or sham (Control) surgery. After 4 wk, LV sections were used for either immunostaining or hematoxylin-eosin staining. Upper panel shows vinculin staining (green) with anti-vinculin antibody and nuclei staining with DAPI (blue). Lower panel shows hematoxylin-eosin staining. *Scale bar*, *10 μm*. (**B**) Isolated cardiomyocytes (CM) were plated on laminin-coated coverslips overnight and then treated with ± 50 nM dasatinib for 24 h. Cells were stained for α-actinin (green) and fodrin (red) with specific antibodies and nucleus with DAPI (blue). *Scale bar*, *10 μm*. (**C**) Cultured cardiomyocytes and CFb, in triplicates, were serum starved overnight and treated with ± 50 nM dasatinib for 30 min. Cells were washed thoroughly and then harvested for cellular uptake of dasatinib by Mass Spectrometry as detailed in the Materials and Methods (n = 3). *p<0.05 vs. cardiomyocytes (CM). (**D**) To study the differential effects of dasatinib on insulin and PDGF induced signaling in cardiomyocytes and CFb, cells were maintained in serum free media overnight in 35 mm culture dishes and then stimulated with either insulin (10 nM) or PDGF (10 ng/mL) for 30 min. For dasatinib treatment, cells were pretreated with the drug (50 nM) 30 min prior to agonist stimulation. Triton-soluble cell extracts were used for Western blots with anti-phospho Akt (Ser473) or anti-phospho ERK1/2 antibodies. Western blot showing GAPDH was used to monitor protein loading. Blots are representative data from three independent experiments.

Dasatinib treatment has been reported to exert cardiac toxicity in a small percentage of patients treated for cancer [27, 28]. Although the concentration used in our studies is substantially lower, we explored whether the administration of this drug at 0.44 mg/kg/day *in vivo* or 50 nM *in vitro* affects cardiomyocyte morphology. Tissue sections from control and TAC mice treated with ±dasatinib were used for actinin and hematoxylin-eosin staining (Fig 6A). These data showed no gross defects in morphology following the *in vivo* administration of dasatinib. Furthermore, similar to our previous measurements [18, 33], we analyzed for changes in the levels of cleaved caspase-3 and calpain in ± dasatinib treated, control and TAC mice by Western blot. These studies revealed that dasatinib treatment either in control or TAC mice did not augment the levels of these markers of programed cell death (data not shown). Our *in vitro* studies using isolated cardiomyocytes showed that dasatinib treatment did not cause significant changes in cell morphology (Fig 6B). Therefore, at a low concentration of dasatinib, sufficient to block NTKs in fibroblasts, dasatinib does not seem to exert any major toxic effects on cardiomyocytes. Next, we analyzed dasatinib uptake by cardiomyocytes and CFb *in vitro*. Using mass spectrometry, the cellular uptake by both cardiomyocytes and CFb after 30 min of dasatinib treatment was measured. When the data was expressed in terms of cell volume, there was a significant increase in the cellular accumulation of dasatinib in CFb when compared to cardiomyocytes (Fig 6C). The higher intracellular concentration of dasatinib could be one of the reasons why CFb are more sensitive to dasatinib treatment than cardiomyocytes. Finally, we analyzed whether dasatinib treatment affected mitogenic signaling preferentially in CFb versus cardiomyocytes. For this, we measured phosphorylation states of Akt and ERK in CFb and cardiomyocytes following stimulation with insulin and PDGF (Fig 6D). Akt and ERK activation is critical for cell survival and proliferation, respectively. Both Akt and ERK activation by PDGF was substantially higher in CFb compared to cardiomyocytes. Dasatinib treatment blocks the activation of both Akt and ERK. In contrast, activation of Akt by insulin, an important survival pathway in cardiomyocytes, was not significantly reduced by dasatinib in either CFb or cardiomyocytes (Fig 6D).

## Discussion

Several studies report a strong correlation between myocardial fibrosis and ventricular dysfunction in patients diagnosed with congestive heart failure (CHF) [1–5]. Importantly, more than one-third of these patients show preserved systolic function, indicating that diastolic dysfunction is a major abnormality in these patients [34]. This maladaptive fibrosis (referred to as “reactive fibrosis”) observed in CHF patients and pressure overload (PO) animal models begins with excessive accumulation of extracellular matrix (ECM) proteins in the perivascular area and then extends to the interstitium of muscle fibers during chronic hemodynamic overload [3]. Recent studies point to CFb as the major cell type involved in interstitial fibrosis [35] and that hyperactivation of these cells contributes to the maladaptive phenotype in the form of a fibrotic, hypertrophied heart [36–39]. Although myocardial fibrosis is considered a major pathological feature associated with ventricular remodeling [40], there are no specific antifibrotic therapeutic options for suppressing cardiac fibrosis in patients with CHF [41, 42]. Therefore, exploring specific signaling pathways within CFb is critical to identify new therapeutic targets to treat patients with CHF. In this context, signaling by receptor and nonreceptor tyrosine kinases has been suggested to be critical for organ fibrosis [11]. Based on our earlier studies that showed the importance of integrin-mediated activation of NTKs in profibrotic signaling in the hypertrophying myocardium [9], we chose dasatinib, a multi-kinase inhibitor that targets primarily NTKs [43], to explore whether blocking tyrosine kinase signaling is sufficient to suppress cardiac maladaptive fibrosis. Dasatinib, under the trade name Sprycel, is used as a cancer medication to treat CML and acute lymphoblastic leukemia (ALL). Long-term dasatinib treatment in CML patients at a dose of 100–140 mg/day is reported to have off-target cardiac toxicities in 7% of patients [27, 28]. However, in a long-term (six years) phase-3 study of CML patients with imatinib-resistance/-intolerance, dasatinib at a 100 mg daily dose was reported to be well tolerated with no new safety issues identified [44]. Our data show that dasatinib delivered by the implanted (i.p.) osmotic pump at a dose of 0.44 mg/kg/day in mice, reduced NTK activation, ECM deposition, hypertrophy and improved LV geometry and function when compared to vehicle treated controls. As mentioned earlier, the dasatinib dose (0.44 mg/kg/day) that we used for our studies in mice corresponds to 2.5 mg for a 70 kg human, based on the formula that takes into consideration both body weight and body surface area [29]. In other words, the dose used in mice is 50 fold lower than the corresponding dose (100–140 mg/day) clinically used for cancer patients. In this context, a recent study describes the use of dasatinib in mice to treat thyroid cancer where a bolus injection (i.p.) of dasatinib at 12.5 mg/kg/day (>25 fold used in our studies) was administered for three weeks with no reported toxicity [45]. Therefore, at appropriately low doses, sufficient to block myocardial tyrosine kinase activation, dasatinib is predicted to be beneficial to the hypertrophied heart.

Our earlier studies using isolated CFb indicated that Pyk2 is the predominant kinase activated during PDGF stimulation [9]. These studies also showed that expression of an inactive form of Pyk2 in cultured CFb reduced ECM accumulation [9]. In the present study, our data showed that: (i) dasatinib treatment blocked both basal and PDGF-stimulated Pyk2 activation and the associated fibronectin secretion and (ii) the expression of Pyk2 mutants (Y402F Pyk2 and Y457F Pyk2) or dasatinib treatment more strongly blocked fibronectin accumulation when compared to the kinase inactive mutants of c-Src and Fak. Other studies have shown that Pyk2 serves as a critical factor in renal fibrosis and has been suggested as a new therapeutic target for ameliorating renal fibrosis [46]. Furthermore, Pyk2 was shown to be required for mechanical stretch-induced activation of ERK1/2 and downstream release of profibrotic factors such as TGFβ and CTGF in renal tubular epithelial cells [46]. Together, these data suggest that Pyk2 might serve as a key NTK for ECM deposition and that the loss of Pyk2 activity might be a primary mechanism for the antifibrotic activity of dasatinib.

To investigate further the mechanism behind the antifibrotic activity of dasatinib, we explored whether the proliferative and migratory potential of CFb were affected by dasatinib treatment. Our earlier work [9] showed that CFb lacking β3 integrin exhibited suppressed ECM deposition, accompanied with loss of cell proliferation and migration. It has been reported that a β3 integrin/PDGFR synergism is required for downstream signaling via NTKs activation. In mitotic cells such as CFb, this synergism might promote proliferation and ECM secretion. Dasatinib has been proposed to be an antifibrotic agent, having its effects on pathways such as PDGFR and NTKs that contribute to fibrosis. Since NTKs are known to function downstream of β3 integrin, we analyzed whether dasatinib treatment in cultured CFb exhibited a similar response; for this, we analyzed nuclear localization of several cell proliferative factors, including Ki67, SKP2 and histone-H2B. Our studies showed that nuclear localization of these factors in CFb that were enhanced during growth factor (PDGF) stimulation, were significantly affected by dasatinib treatment (Fig 4A and 4B). Importantly, histone-H2B was excluded from the nucleus of CFb treated with dasatinib and retained in the cytoplasm. Nuclear localization of histone-H2B has been shown to be critical for cell growth [32]. The loss of tyrosine kinase activity during dasatinib treatment and how it affects the spatial distribution of cell proliferative factors will be the subject of future studies.

As mentioned earlier, the effective dosage of dasatinib was 50 fold lower for these mice when compared to the dosage used clinically in CML patients and therefore, gross morphology of the ventricle was not affected following dasatinib treatment (Fig 6A). It is not clear from our *in vivo* work the actual level of dasatinib in tissues when the mice were given the drug at a sustained dose of 0.44 mg/kg/day; however, in the *in vitro* studies using either cardiomyocytes or CFb, 50 nM dasatinib was an effective low dose that blocked tyrosine kinase signaling and ECM deposition. Further studies are needed to compare whether the 50 nM *in vitro* dose was similar to the dose used for the *in vivo* studies. Earlier studies have shown that treatment of isolated cardiomyocytes with dasatinib at 1 μM for up to 4 days did not significantly affect sarcomere shortening [47]. Compared to the 1 μM dose, the 50 nM dasatinib treatment represents a 200 fold lower concentration, suggesting that is not expected to impact cardiomyocyte contractile functions. Furthermore, our *in vitro* studies revealed that the 50 nM dasatinib treatment did not appear to affect cardiomyocyte morphology.

We also compared cardiomyocytes and CFb for their ability to evoke signaling by two different growth stimulants (insulin and PDGF) in the presence or absence of 50 nM dasatinib. These studies revealed the following: (i) while insulin mediated Akt activation was not significantly affected by dasatinib, (ii) PDGF stimulated Akt activation to a greater extent in CFb and was substantially reduced by dasatinib, (iii) PDGF also stimulated Erk1/2 activation to a greater extent in CFb and that was blocked by dasatinib treatment. Together, these data indicated that PDGF stimulated mitogenic signaling, which occurred more robustly in CFb and was blocked by dasatinib whereas insulin stimulated prosurvival signaling, which is critical for cardiomyocyte survival and was not affected by dasatinib. Lastly, in line with the cell sensitivity data, the cellular uptake studies showed higher accumulation of dasatinib in CFb than in cardiomyocytes. Differential accumulation of drugs has been reported earlier [48] but our study is the first such study with dasatinib in CFb vs. cardiomyocytes. Therefore, at an appropriately low dosage, dasatinib can be expected to suppress pathways responsible for CFb proliferation and ECM deposition without having appreciable effects on cardiomyocyte morphology, function and survival.

In summary, treatment of mice with dasatinib during PO at an effective dose, which was well below the clinically used dosage, was found to offer beneficial effects by reducing cardiac fibrosis and improving ventricular function and geometry. These effects were found to be primarily associated with loss of NTK activity specifically affecting CFb proliferation and migration and ECM deposition.
